# Orthoflavivirus Lammi in Russia: Possible Transovarial Transmission and Trans-Stadial Survival in *Aedes cinereus* (Diptera, Culicidae)

**DOI:** 10.3390/v16040527

**Published:** 2024-03-28

**Authors:** Ivan S. Kholodilov, Sergey V. Aibulatov, Alexei V. Khalin, Alexandra E. Polienko, Alexander S. Klimentov, Oxana A. Belova, Anastasiya A. Rogova, Sergey G. Medvedev, Galina G. Karganova

**Affiliations:** 1 Laboratory of Biology of Arboviruses, FSASI “Chumakov Federal Scientific Center for Research and Development of Immune-and-Biological Products of RAS” (Institute of Poliomyelitis), 108819 Moscow, Russia; polienko.ae@yandex.ru (A.E.P.); mikasusha@bk.ru (O.A.B.); rogova94@icloud.com (A.A.R.); karganova@bk.ru (G.G.K.); 2 Laboratory for the Study of Parasitic Arthropods, Zoological Institute of Russian Academy of Sciences, 199034 St. Petersburg, Russia; s.v.aibulatov@gmail.com (S.V.A.); hallisimo@yandex.ru (A.V.K.); smedvedev@zin.ru (S.G.M.); 3 Laboratory of Biochemistry, FSASI “Chumakov Federal Scientific Center for Research and Development of Immune-and-Biological Products of RAS” (Institute of Poliomyelitis), 108819 Moscow, Russia; aklimentov@mail.ru; 4 Institute of Translational Medicine and Biotechnology, Sechenov First Moscow State Medical University, 119146 Moscow, Russia

**Keywords:** Lammi virus, *Orthoflavivirus*, *Aedes cinereus*, flavivirus, trans-stadial survival, transovarial transmission, mosquitoes

## Abstract

In the last few years, there has been a dramatic increase in the number of discovered viruses that are transmitted by arthropods. Some of them are pathogenic for humans and mammals, and the pathogenic potential of others is unknown. The genus *Orthoflavivirus* belongs to the family *Flaviviridae* and includes arboviruses that cause severe human diseases with damage to the central nervous system and hemorrhagic fevers, as well as viruses with unknown vectors and viruses specific only to insects. The latter group includes Lammi virus, first isolated from a mosquito pool in Finland. It is known that Lammi virus successfully replicates in mosquito cell lines but not in mammalian cell cultures or mice. Lammi virus reduces the reproduction of West Nile virus during superinfection and thus has the potential to reduce the spread of West Nile virus in areas where Lammi virus is already circulating. In this work, we isolated Lammi virus from a pool of adult *Aedes cinereus* mosquitoes that hatched from larvae/pupae collected in Saint Petersburg, Russia. This fact may indicate transovarial transmission and trans-stadial survival of the virus.

## 1. Introduction

In the past few years, there has been a dramatic increase in the number of discovered viruses that are transmitted by arthropods [1,2,3,4]. Some of them are pathogenic for humans and mammals [2,5,6], and the pathogenic potential of others is unknown [1,3,4]. Many recently discovered arthropod viruses belong to the genus *Orthoflavivirus* [1,7,8,9,10].

The genus *Orthoflavivirus* belongs to the family *Flaviviridae* [11] and includes small enveloped viruses with a non-segmented ssRNA(+) genome that encodes one large open reading frame (ORF). The genus *Orthoflavivirus* includes viruses that cause severe human diseases with damage to the central nervous system and hemorrhagic fever [12,13,14,15,16], as well as viruses whose pathogenic potential is unknown and/or insect-specific viruses [17].

The grouping of viruses belonging to the genus *Orthoflavivirus* is well-supported by phylogenetic analyses of their genomic sequences [18,19,20]. According to antigenic properties and associations with vectors and hosts, the genus *Orthoflavivirus* is divided into several groups. Tick-borne virus species are phylogenetically and serologically divided into groups with mammalian and seabird hosts. These viruses are associated with serious human and mammal diseases, including tick-borne encephalitis virus [13], Langat virus [21,22], Powassan virus [23], Louping ill virus [24], Omsk hemorrhagic fever virus [12], and others. Mosquito-borne (*Culex* spp. and *Aedes* spp. vectors) virus species, such as Japanese encephalitis virus, Dengue virus, West Nile virus, yellow fever virus, and Zika virus are causing infections of global concern [14,25,26,27,28,29,30]. Another part of mosquito-borne virus species, such as St Louis encephalitis virus [31] and Murray Valley encephalitis virus [32], are associated with local infections. Viruses with unknown vectors are divided into two groups. The first group includes viruses isolated from bats (Entebbe bat virus group and Rio Bravo virus group), and the second group includes viruses isolated from rodents (Modoc virus group) [33].

The insect-specific virus group can be divided into two lineages [20]. Lineage I represents the classical insect-specific orthoflaviviruses, which were discovered first and are phylogenetically distinct from all other known orthoflaviviruses. In the ICTV taxonomy, they are named related, unclassified insect-specific orthoflaviviruses [17]. Lineage II represents dual-host orthoflaviviruses, which are phylogenetically affiliated with mosquito-borne orthoflaviviruses; therefore, we do not know whether they have vertebrate hosts or whether these viruses have lost the ability to reproduce in vertebrates during their evolution [20]. In ICTV taxonomy, they are named related, unclassified viruses with no known arthropod vector [17]. Most insect-specific viruses were detected in the southern hemisphere [34,35,36,37,38,39,40,41,42,43]. Meanwhile, some were detected in countries in the southern part of the northern hemisphere, including Japan [42,44,45,46,47,48,49,50,51,52,53]. Only four viruses were detected in the northern part of the northern hemisphere: Hanko virus, Ilomantsi virus, Lammi virus in Finland [54,55,56], and Calbertado virus in Canada [57].

Insect-specific viruses are very interesting because many of these viruses are thought to be ancestors to pathogenic arboviruses [58]. Moreover, the primary infection of a vector with an insect-specific virus can block its secondary infection with other viruses. For example, Palm Creek virus has been shown to reduce the infection and replication of the West Nile virus [59]. When West Nile virus superinfects cells primarily infected with Lammi virus, the prior Lammi virus infection restrains the secondary West Nile virus infection [60]. This suggests that the circulation of insect-specific viruses in mosquitoes may influence the spread of mosquito-borne viruses that are already known and pathogenic to humans. The expansion of West Nile virus’ range border to the north has increased the severity of this problem [61]. At the same time, the host’s innate immune system responds differently to viral infections. In a study on the immune response of mosquito cell cultures to infection with insect-specific Lammi and Hanko viruses, it was shown that both viruses caused a strong virus-derived small interfering RNA response, which intensified over time and targeted the whole viral genome. Infection with Lammi virus triggered the production of putative primary piRNAs, while infection with Hanko virus did not [62]. Particular attention should be paid to identifying which species of mosquito is the main vector and host. In different foci of the same viral infection, the role of the vector can be performed by different species [63]. The competence of a mosquito as a biological vector is primarily determined by the possibility for transovarial transmission and trans-stadial survival of the virus.

Mosquitoes are distributed all over the Leningrad region and Saint Petersburg and can be found in most habitats (e.g., forests, parks, etc.). Forty mosquito species are recorded in this region: 25 species of *Aedes*, four species of *Anopheles*, one species of *Coquillettidia*, four species of *Culex*, and six species of *Culiseta* (excluding doubtful records) [64]. *Aedes communis*, *Ae*. *punctor*, *Ae*. *cantans*, *Ae*. *diantaeus*, and *Ae*. *cinereus* predominate in most biotopes of the Leningrad region and Saint Petersburg, and they bite humans from May to September. 

In this work, we studied larvae, pupae, and adult mosquitoes of different species collected in the Leningrad region and Saint Petersburg for the presence of orthoflaviviruses. We detected and isolated Lammi virus from *Ae. cinereus* mosquitoes and showed its potential for transovarial transmission and trans-stadial survival.

## 2. Materials and Methods

### 2.1. Sampling Technique for Mosquitoes

A total of 1396 mosquito specimens (larvae, pupae, and adults) were collected from April to June 2014 in parks within the city of Saint Petersburg (Park Sosnovaya Polyana, (59.831347 N, 30.138416 E); Polezhaevsky park, (59.841955 N, 30.190558 E); and Shungerovskiy Lesopark, (59.836207 N, 30.046358 E)); and in the allotment in the Leningrad Region (Lomonosov District, 59.722681 N, 30.179718 E). Two methods were used to collect mosquitoes. The first method was the collection of biting females with a Krishtal glass trap from human bodies [65]. Briefly, the Krishtal trap is a glass ball (diameter 10 cm) with two tubes (one long, the other short) located at an angle of 90 degrees to each other. The long tube is used to hold the trap, and the short tube is used to cover the mosquito. Inside the glass ball, a short tube forms a “skirt” that prevents the insect from flying back out (Figure S1). The second method was the collection of larvae and pupae from temporary and permanent water bodies. The sampling techniques for mosquitoes were reviewed previously [66]. Briefly, mosquito larvae and pupae were sampled with a dipper sieve with a 20 cm diameter, sweeping it two times through the surface water layer. The collected larvae were removed from the sieve in plastic cuvettes and transferred to 1 L containers together with water from the breeding site. In the laboratory, a room with an average daily air temperature of 15 °C was used for mass rearing. The larvae were placed in groups of 10–30 individuals in 2 L containers. These containers were checked daily during the development of larvae to detect pupae; the water was changed every three days; and the larvae were fed with crushed daphnia. Pupae were transferred to 500 mL containers covered with mosquito netting.

Adult mosquitoes (collected in nature or after hatching) were placed in a freezer at a temperature of −20 °C for five minutes. Immediately after this, we identified mosquito specimens by using available keys [67,68,69]. After identifying the specimens of mosquitoes, they were placed in tubes and frozen at −80 °C.

### 2.2. Preparation of Mosquito Suspensions 

Adult mosquitoes (male, female, or male+female) were homogenized in pools of 15–17 specimens according to species, location, and route of collection using the TissueLyser II laboratory homogenizer (QIAGEN, Hilden, Germany) in 0.9% saline solution (FSASI Chumakov FSC R&D IBP RAS, Moscow, Russia). After homogenization, mosquito suspensions were centrifuged at 1500 rcf for 5 min. The supernatant was used to isolate total RNA.

### 2.3. Detection of Orthoflaviviruses by RT-PCR and Sequencing

Total RNA from mosquito suspensions and infected cell culture supernatant was isolated with TRI Reagent LS (Sigma-Aldrich, St. Louis, MO, USA), according to the manufacturer’s protocols. Reverse transcription was performed with random hexamer primer (R6) and the MMLV reverse transcriptase kit (Promega, Madison, WI, USA), according to the manufacturer’s protocols. PCR was performed using cDNA, virus-specific oligonucleotides, and DreamTaq DNA polymerase (Thermo Fisher Scientific, Vilnius, Lithuania), according to the manufacturer’s protocols. To detect orthoflaviviruses in mosquitoes, we used the primers described earlier [70]. To detect Lammi virus in infected cell culture supernatant, we used the following primers: Lm9840F—5′-GCACCATTTCCATAAGTTATC-3′ and Lm10270R—5′-GACTGACACACGTATGTTATC-3′ (genome locus NS5, amplicon size 472 bp, temperature 50 °C). To obtain the complete genome of Lammi virus we used the following primers: Lm20F—5′-AGTATATTCTACGTGTGCGTT-3′ and Lm1615R—5′-GAAGCGCTAGATCTTGGTACC-3′ (5′NTR—E, 1595 bp, 50 °C); Lm1410F—5′-ATTTCCATTCACGGACAGTCT-3′ and Lm3000R—5′-CCCAGCAACTCCGTGTCACAG-3′ (E—NS1, 1631 bp, 50 °C); Lm2740F—5′-AGCGTGGTTGTGAAGAATGC-3′ and Lm3740R—5′-CCTCCAGTGTTCATCTCTGC-3′ (NS1—NS2A, 1016 bp, 50 °C); Lm3620F—5′-GAGACGCATGACAAGCAAGTAC-3′ and Lm4740R—5′-GTGTGACGTGCCACATTGTGTG-3′ (NS2A—NS3, 1160 bp, 50 °C); Lm4320F—5′-GCGGCCGCCTCATTGATATTCG-3′ and Lm5770R—5′-TTGGATACTCGTCGTTAAATG-3′ (NS2B—NS3, 1486 bp, 50 °C); Lm5670F—5′-TCGAGTGGATAACAGATTACG-3′ and Lm6580R—5′-TCTATAGCCAACCGATAAGC-3′ (NS3—NS4A, 949 bp, 50 °C); Lm5940F—5′-GAGAGAGTGGTTCTAGGAAC-3′ and Lm7135R—5′-GTCCATGGAAAGCAATATTCC-3′ (NS3—NS4B, 1236 bp, 50 °C); Lm6850F—5′-AACGATCAGTCCAAGACAAC-3′ and Lm7800R—5′-GTCCGGTCAACTTCTGTTATTCC-3′ (NS4A—NS5, 952 bp, 50 °C); Lm7600F—5′-CTCATCAACGGTATGGAATACG-3′ and Lm9300R—5′-AGGTCAGCATTGGTTATTCTG-3′ (NS4B—NS5, 1776 bp, 50 °C); Lm9060F—5′-AATATGATGGGAAAGCGTGAAAA-3′ and Lm9980R—5′-TCACGGCGGTGGAAGTGAATG-3′ (NS5, 965 bp, 50 °C); Lm9780F—5′-AGACATCAATGAGTGGAGAGCT-3′ and Lm10360R—5′-ATTGTGAATTCAGCTGGAATGCT-3′ (NS5, 627 bp, 50 °C); Lm10120F—5′-GGACAACATGGTCAATACATG-3′ and Lm10700R—5′-AGTTACTTGCTGTTTTACAACC-3′ (NS5—3′NTR, 664 bp, 50 °C); Lm8400F—5′-CCTCACTAAGCAGGACACTGCT-3′. The obtained PCR products were analyzed in agarose gel, with bands of the target length being extracted from the gel. The bands were purified using the QIAquick Gel Extraction Kit (QIAGEN, Hilden, Germany) and sequenced with the Applied Biosystems 3500 genetic analyzer (Waltham, MA, USA) using the BigDye Terminator v3.1 Cycle Sequencing Kit (Thermo Fisher Scientific, Vilnius, Lithuania). The obtained sequences were analyzed using Lasergene® SeqMan Pro Software Version 7.0.0 (DNASTAR Inc., Madison, WI, USA).

### 2.4. Isolation of Lammi Virus on Cell Culture

The cell culture C6/36 (*Aedes albopictus*) was infected with a mosquito suspension positive for the presence of orthoflavivirus RNA in PCR or with an infected culture supernatant. One hundred microliters of the mosquito suspension or infected culture supernatant were added to flat-sided culture tubes (Nunc, ThermoFisher Scientific, Waltham, MA, USA) containing the cell culture and incubated for 1 h at 26 °C. After this, 2 mL of a maintenance medium, consisting of L-15 (Leibovitz) medium (FSASI Chumakov FSC R&D IBP RAS, Moscow, Russia), 2% fetal bovine serum (Gibco, Paisley, UK), and antibiotics (100 U/mL penicillin, 100 μg/mL streptomycin), were added, and the mixture was incubated at 26 °C for 7 days. The infected culture supernatant was harvested immediately after the appearance of cytopathogenic effect (CPE) or on the 7th day after infection in the absence of CPE.

### 2.5. Phylogenetic Analysis

The RNA sequences of some representatives of the genus *Orthoflavivirus* and the strains described in this article were used in phylogenetic analysis. The nucleotide sequences of the complete ORF were aligned using ClustalW. To identify ORFs, we used the Snap Gene Viewer program, with translation options set to a minimum length of 75 amino acids and selecting the options “Require a start codon ATG”, “except at DNA ends”, and “Standard” of the genetic code for ORFs and new features. Phylogenetic analysis was conducted using the maximum likelihood method and the Tamura–Nei model [71] in MEGA X with 1000 bootstrap replications [72].

## 3. Results

### 3.1. Collection of Mosquitoes and Lammi Virus Detection

From April to June 2014 in Saint Petersburg and the Leningrad Region, eight species of four mosquito genera were collected: *Ae. cinereus*, *Ae. cantans*, *Ae. communis*, *Ae. diantaeus*, *Ae. punctor*, *Culiseta morsitans*, *Anopheles claviger*, and *Culex territans*. In total, 1396 individuals were caught, of which 128 adults were collected from the human body by Krishtal trap, and 308 pupae and 960 mosquito larvae were collected from temporary and permanent water bodies. Out of the 960 mosquito larvae caught, 653 larvae developed to adults, and 44 larvae developed to pupae. Out of the 308 mosquito pupae caught, 264 pupae developed to adult mosquitoes (Table 1). Thus, 1045 adult mosquitoes, 88 pupae, and 263 larvae were combined into 177 pools and homogenized, and the homogenates were screened for the orthoflavivirus NS5 RNA. The RT-PCR product from one orthoflavivirus-positive adult mosquito pool, obtained from *Ae. cinereus* larvae/pupae collected in Park Sosnovaya Polyana of Saint Petersburg, was Sanger-sequenced. The resulting sequence was analyzed with BLAST. The resulting fragment was similar to that of the previously described Lammi virus (NC024806, FJ606789) [54,55], with 99.11% identity.

### 3.2. Isolation of Lammi Virus in Cell Line

To isolate the virus from the mosquito homogenate, we infected the C6/36 cell line. After infection, the C6/36 cell line was kept at 26 °C. CPE appeared on the 3rd day after infection. Supernatants from infected cell cultures tested positive by RT-PCR for the presence of Lammi virus RNA. All positive samples were confirmed by sequencing.

### 3.3. Phylogenetic Analysis and Genomic Identity Assessment

**Figure 1:**
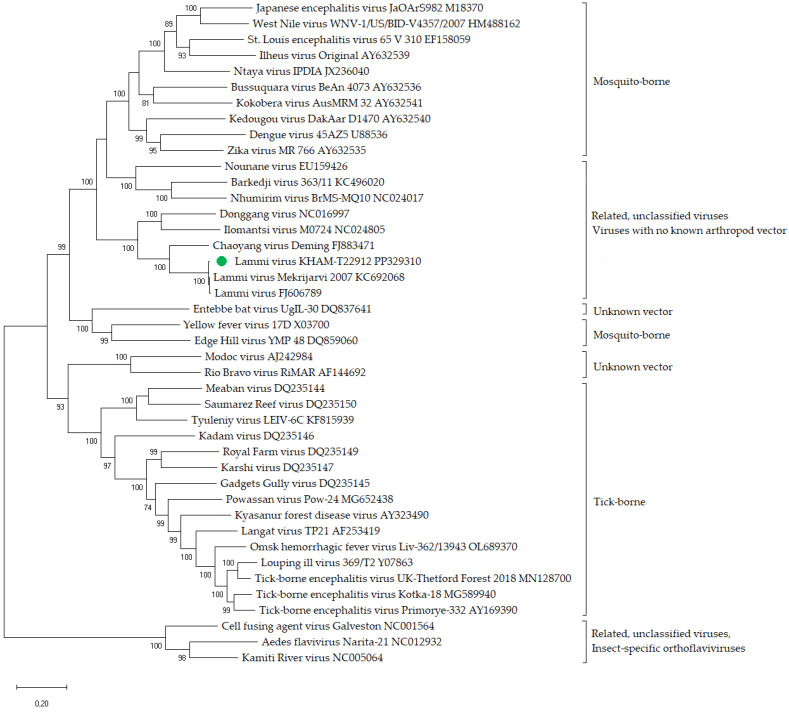
Phylogenetic analysis of representatives of the genus *Orthoflavivirus*. Phylogenetic trees were constructed using sequences of the complete open reading frame in MEGA X with the maximum likelihood method (1000 bootstrap replications). Bootstrap values (>70%) are shown at the branches. GenBank accession numbers are listed for each strain. Green circle—strain of Lammi virus described in this study.

According to the phylogenetic analysis conducted using sequences of the complete ORF, strain KHAM-T22912 clustered with other strains of Lammi virus formed one monophyletic group (Figure 1).

The nucleotide and amino acid identities of Lammi virus strains were assessed using the complete ORF. The nucleotide identity of the strain KHAM-T22912 compared to Lammi virus (FJ606789) and strain M0719 were 98.49% and 98.88%, respectively. The amino acid identities of strain KHAM-T22912 compared to Lammi virus (FJ606789) and strain M0719 were 99.86% and 99.89%, respectively.

## 4. Discussion

The investigation of viromes across various arthropods, including ticks, mosquitoes, sandflies, etc., using high-throughput sequencing has led to the discovery of a large number of viruses, most of which can be classified as insect-specific viruses [1,4,73,74,75]. These viruses belong to different families and orders [73,76], naturally infect arthropods, and replicate in arthropods and/or insect cell lines, but their distinctive feature is their inability to replicate in vertebrates and their cells [73]. Although insect-specific viruses do not cause disease in mammals, they can play an important role in interactions with pathogenic arboviruses. Insect-specific viruses can increase [77], reduce [60,78], or have no effect [79] on the reproduction of known arboviruses that are pathogenic to mammals. 

As mentioned above, insect-specific viruses of the genus *Orthoflavivirus* can be divided into two lineages [20]. Lineage I represents the viruses that were discovered first and are phylogenetically distinct from all other known orthoflaviviruses, and lineage II represents dual-host orthoflaviviruses, which are phylogenetically affiliated with mosquito-borne orthoflaviviruses [17].

Lammi virus is a representative of the dual-host orthoflaviviruses. It was first detected in an adult *Aedes* spp. mosquito pool obtained from individual homogenates of mosquitoes in Finland in 2004 [55]. The species of Lammi-positive mosquito was identified according to mitochondrial cytochrome *C* oxidase 1 DNA sequences. The sequences were found to be 99% identical in the alignable region with the *Ae. cinereus* cytochrome *C* oxidase 1 sequence [55]. Later, Lammi virus was detected in a mosquito pool in Finland in 2007 [54]. The species of mosquitoes, as in the previous case, were identified according to mitochondrial cytochrome *C* oxidase 1 DNA sequences. As the mosquitoes were homogenized in pools, PCR product clones were obtained to determine the species. According to phylogenetic analysis, the Lammi-positive pool contained the following mosquitoes: *Aedes riparius*, *Ae. punctor*, *Ae. annulipes*, and *Ae. cantans* [54]. It is still unclear whether Lammi virus is an arbovirus or a mosquito-specific virus. Therefore, it is necessary to determine its main reservoir and vector to understand its possible spread and host range.

In our work, Lammi virus was first detected in Russia in adult *Ae. cinereus* mosquitoes molted from larvae/pupae that were collected from temporary or permanent reservoirs in Saint Petersburg. We did not detect Lammi virus in other mosquito species. This may have been due to the insufficient number of studied mosquitoes or the fact that transovarial transmission and/or trans-stadial survival of this virus was not observed in other mosquito species.

*Aedes cinereus* mosquitoes, from which Lammi virus was isolated, are widely distributed and populate Northwestern Russia [64,80], Western Europe, European Russia, Siberia, the Russian Far East (including Sakhalin Island and the Kamchatka Peninsula), Transcaucasia, Central Asia, and North America [68,69]. In Western Europe, *Ae. cinereus* can be found in the UK, Belgium, the Netherlands. Norway, Sweden, Finland, Estonia, Latvia, France, Italy, Bulgaria, and Turkey [81]. The larvae of *Ae. cinereus* usually hatch at a temperature of 12–13 °C, and their development starts at 14–15 °C [82], and they can be found from April to August in different water bodies (ponds, backwaters of rivers, puddles, etc.) [83]. The females bite humans and other mammals mainly at dusk and dawn, from May to September [67,84]. The females of *Ae. cinereus* differ from those of other *Aedes* species due to their short proboscis, and the males have genitalia with a divided style [67,68,69]. However, *Ae. rossicus* and *Ae. geminus* are externally similar to *Ae. cinereus*; these species are difficult to identify and can be reliably determined only by male genitalia. 

According to the phylogenetic analysis, strain KHAM-T22912 from Russia forms one monophyletic group with strains from Finland. All Lammi virus strains were isolated in different years and regions, but their nucleotide and amino acid identities are very high. This may indicate the low variability of the virus.

Previously, Lammi virus was isolated in the C6/36 (*Aedes albopictus*), AA23 (*Ae. albopictus*), and A20 (*Ae. aegypti*) cell lines. The incubation temperature was below 30 °C [54]. Strain KHAM-T22912 from Russia was isolated in C6/36 at 26 °C. We did not use any mammalian cell lines for isolation, as it was previously shown that Lammi virus did not reproduce in mammalian cell lines, such as primary chicken, human (SH- SY5Y, HEK293, HeLa, Hep, MRC-5, SW13, HEK293), mouse (Neuro2A, L929), hamster (BHK-21), porcine (PK-15), monkey (Vero, VeroE6, BGM and MA104), canine (MCDK), and toad (XTC) cell lines [54,55].

Since, in our work, Lammi virus was isolated from adult *Ae. cinereus* mosquitoes molted from larvae/pupae, this may indicate that the virus is able to survive trans-stadially and most likely can be transmitted transovarially. *Ae. cinereus* mosquitoes may serve as reservoirs for Lammi virus.

This work and previous works [55] may serve as evidence that the host of Lammi virus is the *Ae. cinereus* mosquito, and we can expect that this virus also circulates in other areas where these mosquitoes are present. 

## 5. Conclusions

We have isolated Lammi virus from *Ae. cinereus* mosquitoes in the European part of Russia and showed its potential for transovarial transmission and trans-stadial survival.

## Figures and Tables

**Table 1:** Mosquito species and their collection locations.

Mosquito Species	Mosquito Stage at Collection	Mosquito Stage in Study (Gender)	Number of Individuals
Saint Petersburg, Park Sosnovaya Polyana (59.831347 N, 30.138416 E)			
*Aedes cinereus*	larvae/pupae	adult (m+f)	19 *
	larvae	adult (m+f)	21
		larvae	51
*Aedes cantans*	adult	adult (f)	4
	larvae/pupae	adult (m+f)	3
	pupae	adult (m/f)	171
		pupae	44
	larvae	adult (m/f)	68
		larvae	5
*Aedes communis*	adult	adult (f)	7
	pupae	adult (m+f)	21
	larvae	adult (m+f, f)	87
*Aedes punctor*	pupae	adult (f)	3
	larvae	adult (f)	6
*Aedes dianteus*	larvae	adult (f)	1
*Aedes* spp.	larvae	pupae	4
		larvae	70
**Saint Petersburg, Polezhaevsky park (59.841955 N, 30.190558 E)**			
*Aedes cinereus*	adult	adult (f)	4
	larvae/pupae	adult (f)	4
	pupae	adult (m+f)	12
	larvae	adult (m/f)	62
*Aedes cantans*	adult	adult (f)	90
*Aedes communis*	adult	adult (f)	5
	pupae	adult (m+f)	10
	larvae	adult (m+f)	11
*Aedes punctor*	adult	adult (f)	4
*Aedes dianteus*	larvae	adult (f)	6
*Aedes* spp.	larvae	larvae	60
**Saint Petersburg, Shungerovskiy Lesopark (59.836207 N, 30.046358 E)**			
*Aedes communis*	adult	adult (f)	12
	pupae	adult (m)	38
	larvae	adult (m/f)	287
		larvae	22
*Aedes punctor*	adult	adult (f)	2
	larvae	adult (m/f)	13
		larvae	30
*Aedes* spp.	larvae	pupae	40
**Leningrad Region, Lomonosov District (59.722681 N, 30.179718 E)**			
*Aedes cinereus*	larvae	adult (m+f)	28
*Culex territans*	larvae	adult (m+f)	24
		larvae	25
*Anopheles claviger*	pupae	adult (f)	2
*Culiseta morsitans*	larvae	adult (m+f)	20
**Total:**			**1396**

* This pool was positive for Lammi virus. m+f—mosquito pool with both males and females. m/f—mosquito pools that contain only males or only females.

## Data Availability

The data presented in this study are available in the article and Supplementary Material. The obtained sequencing data were deposited in the GenBank database, under Lammi virus, strain KHAM-T22912 (PP329310).

## Supplementary Materials

The following supporting information can be downloaded at: https://www.mdpi.com/article/10.3390/v16040527/s1, Figure S1: Krishtal trap.
