# Supplementary figures and images for: Orthoflavivirus Lammi in Russia: Possible Transovarial Transmission and Trans-Stadial Survival in Aedes cinereus (Diptera, Culicidae)
Source: Viruses. 2024 Mar 28;16(4):527. doi: 10.3390/v16040527 (PMC11054007; doi:10.3390/v16040527)

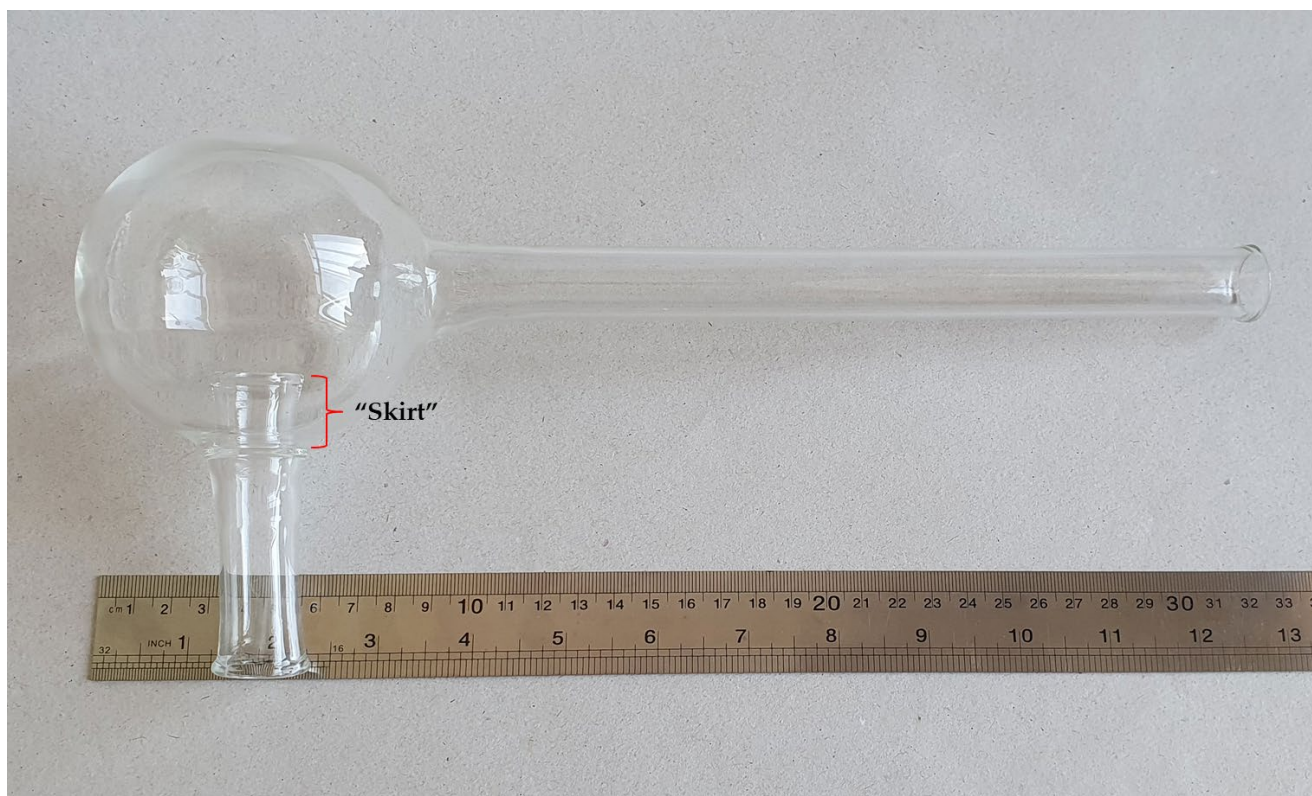

**Figure S1.** Krishtal trap.

Supplement: Supplementary file 1 [file viruses-16-00527-s001.zip › viruses-2893841-supplementary.pdf]
